# Comparison of three models fitting the soil water retention curves in a degraded alpine meadow region

**DOI:** 10.1038/s41598-019-54449-8

**Published:** 2019-12-05

**Authors:** Tao Pan, Shuai Hou, Yujie Liu, Qinghua Tan

**Affiliations:** 1 Key Laboratory of Land Surface Pattern and Simulation, Institute of Geographic Sciences and Natural Resources Research, Chinese Academy of Sciences, Beijing, 100101 China; 2 Institute of crop science, Chinese Academy of Agricultural Sciences/Key laboratory of crop physiology and ecology, Ministry of Agricultural and Rural Affairs, Beijing, 100081 China

**Keywords:** Hydrology, Solid Earth sciences

## Abstract

Soil water retention curve (SWRC) plays an important role in simulating soil water movement and assessing soil water holding capacity and availability. Comparison of fitness between different models to determine the best SWRC model of specific regions is required. In this study, three popular models, van Genuchten, Brooks Corey and Gardner model, were selected for comparing in a degraded alpine meadow region on the eastern Tibetan Plateau. Fitness, error distribution along with key parameters were compared. For each soil horizon, the soil moisture content at all soil water potentials decreased consistently with degradation, thereby integrally moving the SWRCs of all soil depths downward with degradation. The differences in SWRCs across various degradation degrees diminished along with soil depth and soil water potential. The Adj.*r*^2^ values of van Genuchten, Brooks Corey and Gardner models ranged in 0.971–0.995, 0.958–0.997, and 0.688–0.909, respectively. The van Genuchten and Brooks Corey models significantly (*p* < 0.05) outperformed the Gardner model, and have no significant differences in fitness. The fitness of all three models showed no significant changes with degradation. Regardless of degradation degree and soil depth, the fitting error of van Genuchten and Brooks Corey models was mainly distributed in the higher (from –100 hPa to –500 hPa) and lower (below –10000 hPa) potential sections. With regard to the parameters of van Genuchten and Brooks Corey models, the field capacity (*θ*s), and permanent wilting moisture were highly coherent with Adj.*r*^2^ values of higher than 0.98, while the curve shape parameter (*θ*r), and air entry pressure of the Brooks Corey model were much lower than those of the van Genuchten model with Adj.*r*^2^ values of lower than 0.91. The SWRCs with varying degrees of degradation are best fitted by both van Genuchten and Brooks Corey models but cannot be fitted by Gardner model. Soil water holding capacity decreased with degradation especially in the top soil (0 cm to 30 cm), but the curve shape of all SWRCs did not change significantly with degradation.

## Introduction

Filled in soil pores and absorbed by soil particles, soil water sustains vegetation and lies in the center of a terrestrial hydrologic cycle^1,2^. From the perspective of work–energy theorem, soil water flow and retention are essentially controlled by a potential gradient. The soil water retention curve (SWRC) illustrates the relationship between soil water content and soil water potential^3^. SWRC not only includes abundant information about the physical and hydraulic properties of soil but also influences root uptake and evaporation^4–6^. Therefore, SWRC is considered one of the most fundamental soil hydraulic properties^7^, and the accurate acquisition of SWRC and its parameterization have great significance in understanding soil moisture dynamic and soil hydrology^8^.

There are two main approaches to obtaining SWRCs: the first is experimental determination and the second is derivation from basic soil properties by using PTFs (pedotransfer functions)^9,10^. Although experimental approach is time consuming and costly, it is undoubtedly more precise and reliable for SWRCs of specific soils. To fit the discrete measured data, an array of empirical or semi-empirical models have been developed and revised^7,11^. Among them, some models (e.g. van Genuchten, Brooks Corey and Campbell model) have explicit physical significance and have showed their feasibility for a wide variety of soils^12–14^. SWRCs of different soils might vary remarkably due to the high spatial heterogeneity of soil properties, while the applicability of each model is limited by its certain curve-shape characteristics^15^. For example, Roy *et al*.^16^ used the van Genuchten model to developed SWRCs for three soils in the Red River Valley, and found that the silt and sandy loam soils had the best fitted SWRCs in terms of shape and slope while the silty clay soil did not show a good match. Similarly, Shervin *et al*.^15^ compared the applicability of SWRCs using three major soil samples with different soil textures and from different geographical locations within Iran, indicating that the applicability of SWRCs varied owing to different soil texture. Therefore, model selection and comparison are prerequisite before the most suitable one can be determined for SWRCs of specific soils, and suchlike comparisons have been conducted^11,17,18^.

Due to climate change and human activities, alpine ecosystems on the globe have been undergoing remarkable degradation^19,20^. Given that alpine regions are usually headwaters, the subsequent effect of degradation on alpine soil hydrology has received increasing attention^21,22^. The Tibetan Plateau is the headwaters region of the Yangtze, Yellow and Mekong rivers, which are the world’s third, fifth and seventh longest rivers, respectively. The meadows in Tibetan Plateau have been significantly degraded due to the influences of climate change, overgrazing, human activities and rodents in the past decades^23^, which impacts soil physical and chemical properties, hence influencing soil hydraulic properties as well as soil moisture conditions^24^. Studies on the influence of degradation on soil water content and hydrological properties have been carried out^25,26^. Pan *et al*.^27^ showed that the soil moisture content and field capacity decreased consistently with the alpine degradation. Yang *et al*.^28^ investigated soil moisture content covering potential between 0 to −15000 hPa among different soil pedogenic horizons. The study of Wang *et al*.^29^ showed that the Gradner model had a good simulation of the SWRCs with alpine grassland degradation in the source area of the Yellow River. However, comparisons of models for SWRCs carried out by Bayat *et al*.^30,31^ showed that the Gardner model was not an appropriate model for the soils because of its low fitting accuracy. Because literature about SWRCs fitting and comparison in alpine region in Tibetan Plateau still remains relatively scarce compared to those in low-altitude-areas, the applicability of meaningful and widely accepted models for the alpine soil in the Tibetan Plateau, especially for degraded alpine/meadows, remains unknown, which hinders understanding the water regime and dynamic of alpine soil. Therefore, exploring and comparing the applicability of models in fitting the SWRCs of degraded alpine meadow soil warrants further research.

Based on detailed field investigation, a series of plots representing differently degraded alpine meadow were chosen on the east edge of the Tibetan Plateau, and three popular fitting models were selected to fit the SWRCs of those plots. The objectives of this study are (1) to analyze the influence of alpine meadow degradation on SWRCs, (2) to evaluate the fitness of some popular models for the SWRCs of degraded alpine meadow, and (3) to characterize the fitness and fitting errors of different models across varying degradation degrees.

## Materials and Methods

### Study area, degradation classification and basic soil properties

**Figure 1:**
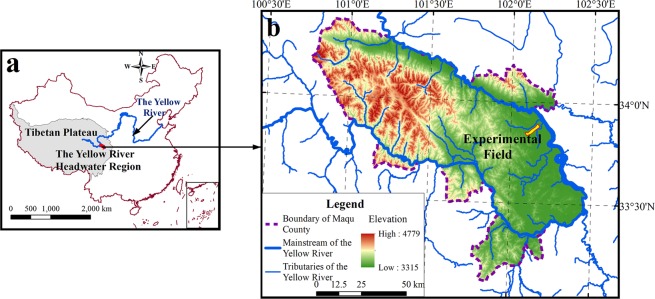
Location of the study area and experimental field. The map was generated by ArcGIS 10.2 software^54^.

The study area is located in the Roige Wetland on the eastern edge of the Tibetan Plateau (100°50′–102°30′E, 33°10′–34°30 N) and the upper reaches of the Yellow River with an average elevation of about 3800 m above sea level (Fig. 1a). The area has a frigid humid monsoon climate with annual mean temperature and precipitation of approximately 1.2 °C and 600 mm, respectively^32^. The main soil types in this area include silt loam and sandy loam, while its vegetation is mainly alpine meadows dominated by *kobresia*^33^.

An area of about 2 km^2^ that encloses the flat grassland across the terrace of the Yellow River was selected as the experimental field (102°12′45′′E, 33°46′28′′N; 3435 m above sea level) (Fig. 1b). The area has been experiencing degradation because of overgrazing. The vegetation characteristics, including vegetation coverage, dominant species, number of species, and above ground/underground biomass, were used as indicators to assess the degradation degree^34^, and the degrees of degradation in the study area was identified as light, moderate, and severe (Table 1). Because of variation in grazing intensity, rodent activities and topographic conditions, patches of grassland showing evidence of initial degradation to almost complete barrenness have emerged across the field, we chose sites in various degrees of degradation in small areas to represent the degradation of the study area using the space-for-time substitution strategy^34,35^. After a detailed field investigation during the summer of 2014, nine plots were selected and each degradation degree equally embraced three plots. The average plant height in the field ranges between 10 cm and 15 cm.

**Table 1:** Vegetation characteristics of three alpine meadows with varying degradation degrees.

Degradation degree	Vegetation coverage (%)	Number of species	Dominant species	Above/below ground biomass (g·m^–2^)
Light	80.5 ± 4.9	18–25	*Kobresia tibetica*, *Kobresia humilis*, *Stipa aliena*	416 ± 43/2149 ± 47
Moderate	59.7 ± 4.5	15–20	*Kobresia pygmaea*, *Agropyron cristatum*, *Carex tristachya*	201 ± 70/1929 ± 154
Severe	13.7 ± 8.6	5–12	*Kobresia robusta*, *Leymus chinensis*, *Potentilla bifurca*	52 ± 39/842 ± 91

**Figure 2:**
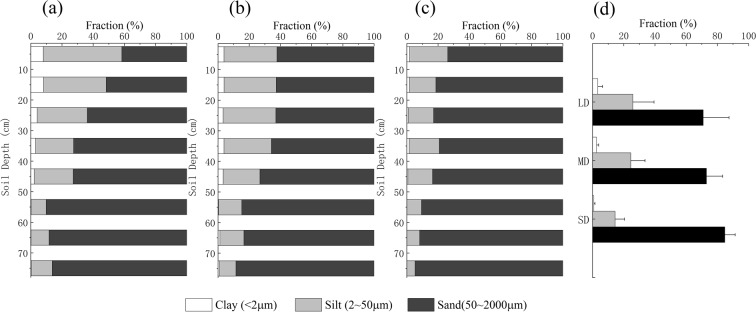
Particle size distributions of light (**a**), moderate (**b**), severe degradation (**c**) and average (**d**) soil samples corresponding to soil texture classifications.

Undisturbed soil samples were collected 0 cm to 80 cm deep at each plot with an interval of 10 cm for SWRCs measurement. Table 2 lists the basic soil properties, including bulk density, soil organic carbon, and soil texture. The bulk density increased and the soil organic carbon decreased along with increasing degradation degree. As the particle size distributions of light (LD), moderate (MD), and severe degradation (SD) soil samples corresponding to soil texture classifications showed in Fig. 2, the statistical analysis revealed that significant changes mainly took place in the upper soil layers (0 cm–30 cm) along with the degradation. The sandification of the soil along with its degradation can be clearly seen from the soil texture fraction (Fig. 2).

**Table 2:** Bulk density and soil organic carbon of all soil samples. The letters that follow the values denote a significant difference (p < 0.05) between the degradation degrees.

Depth (cm)	Bulk Density (g·cm^−3^)			Soil Organic Carbon (g·kg^−1^)		
	Light	Moderate	Severe	Light	Moderate	Severe
0–10	0.83 ± 0.08a	0.98 ± 0.07b	1.09 ± 0.04c	58.45 ± 11.85a	45.32 ± 8.02b	14.78 ± 8.67c
10–20	0.91 ± 0.08a	1.00 ± 0.10b	1.12 ± 0.03c	39.97 ± 12.56a	29.22 ± 9.21b	10.43 ± 5.38c
20–30	1.05 ± 0.11a	1.05 ± 0.08a	1.14 ± 0.05b	26.51 ± 11.80a	25.12 ± 9.75a	9.20 ± 3.01b
30–40	1.17 ± 0.04a	1.14 ± 0.11a	1.15 ± 0.08a	15.69 ± 3.27a	11.92 ± 3.29b	7.57 ± 1.18c
40–50	1.26 ± 0.06a	1.18 ± 0.06a	1.23 ± 0.03a	11.62 ± 4.05a	11.29 ± 5.03a	6.95 ± 1.13b
50–60	1.27 ± 0.03a	1.22 ± 0.19a	1.29 ± 0.04a	9.09 ± 3.15a	6.46 ± 2.61b	6.38 ± 0.93b
60–70	1.33 ± 0.07a	1.42 ± 0.14a	1.41 ± 0.14a	6.85 ± 1.63a	7.57 ± 2.68a	6.17 ± 1.32a
70–80	1.41 ± 0.08a	1.44 ± 0.02a	1.46 ± 0.10a	6.72 ± 0.79a	6.78 ± 2.07a	5.86 ± 1.31a

### Measurement of SWRCs

The current methods for obtaining SWRCs is mainly determined on experiment. The measurement principle is the soil water content at different soil water potentials based on the dehumidification process. A pressure plate apparatus (1500 F1, Soil Moisture Equipment Corp., SEC, U.S.) was used to measure the soil water content at different soil water potentials to determine the SWRCs^36^. The gravimetric water content at 10, 50, 100, 200, 300, 500, 1000, 3000, 5000, 7000, 10000, 15000 hPa was measured using the oven-dried method (105 °C, 12 h). The volumetric water content (*θ*) was calculated by multiplying gravimetric water content by bulk density.

The saturated water content was also measured. Metal cylinder cores (50.46 mm in diameter and 50 mm in height) with undisturbed soil samples were soaked in 4.8 cm deep water until constant weight was reached, and the volumetric water content was calculated by determining the gravimetric water content^37^. Soil water potential corresponds to saturated water content and can be rated as zero. With the soil water content at 12 pressures and the saturated water content (0 hPa), 13 soil water potentials ranging from 0 hPa to 150000 hPa were obtained for each soil sample.

### Three fitting models

The SWRC is derived by fitting the acquired paired data, including the measured soil moisture content and the corresponding preset soil water potential. We used the van Genuchten, Brooks Corey, and Gardner models to fit the acquired data using the RETC software (Salinity Laboratory, USDA)^38^. The van Genuchten model is expressed as follows^39^:

1 $$\frac{{\rm{\theta }}-{{\rm{\theta }}}_{{\rm{r}}}}{{{\rm{\theta }}}_{{\rm{s}}}-{{\rm{\theta }}}_{{\rm{r}}}}={(1+{(\alpha |h|)}^{{\rm{n}}})}^{-{\rm{m}}},\,{\rm{m}}=1-\frac{1}{{\rm{n}}}$$

where *θ* is the soil moisture content (Vol., %), while *θ*_s_ and *θ*_r_ are the saturated soil moisture content (Vol., %) and residual soil water content (Vol., %), respectively. The left part of the equation denotes the effective water saturation. *h* denotes the soil water potential (hPa, also written as cm (H_2_O)), *α* is the scaling parameter which reciprocal can be rated as the air entry pressure (cm^−1^), and *n* is a dimensionless parameter related to curve shape.

The Brooks Corey model can be written as follows^40^:

2 $$\frac{{\rm{\theta }}-{{\rm{\theta }}}_{{\rm{r}}}}{{{\rm{\theta }}}_{{\rm{s}}}-{{\rm{\theta }}}_{{\rm{r}}}}=\{\begin{array}{c}{(\frac{{\rm{h}}}{{{\rm{h}}}_{{\rm{d}}}})}^{\lambda },h > {{\rm{h}}}_{{\rm{d}}}\\ 1,h < {{\rm{h}}}_{{\rm{d}}}\end{array}$$

where the left part of the equation denotes the effective water saturation (the same as Eq. 1), *h* denotes the soil water potential (cm), *h*_d_ denotes the air entry pressure (cm), and *λ* is a dimensionless parameter related to curve shape.

The Gardner model is expressed as follows^41^:

3 $${\rm{\theta }}={\rm{a}}{|{\rm{h}}|}^{-{\rm{b}}}$$

where *θ* denotes the soil moisture content (Vol., %), *h* denotes the soil water potential (cm), and *a* and *b* are two fitting parameters. This model is much simpler than the former two models.

### Fitness assessment and comparison

The fitness of the three models is assessed using the adjusted coefficient of determination (Adj.*r*^2^), root mean square error (RMSE), and relative error (RE)^42,43^. Adj.*r*^2^ is computed as follows:

4 $${\rm{Adj}}.{{\rm{r}}}^{2}=1-\frac{{\rm{N}}-1}{{\rm{N}}-{\rm{k}}-1}(1-{{\rm{r}}}^{2})$$

where *N* − 1 is the degree of freedom (*d*.*f*.) of the sample, *k* is the number of explaining variables, *N* − *k* − 1 is the *d*.*f*. of the residual error, and *r*^2^ is the coefficient of determination. The value of Adj.*r*^2^ ranges from 0 to 1, with a higher value indicating a better fitness.

RMSE and RE are computed as follows:

5 $${\rm{RMSE}}=\sqrt{\frac{\sum {({\rm{y}}-{\rm{y}}^{\prime} )}^{2}}{{\rm{N}}}}$$

6 $${\rm{RE}}=\frac{|{\rm{y}}-{\rm{y}}^{\prime} |}{{\rm{y}}}\times 100 \% $$

where *y* and *y*′ are the measured and fitted values, respectively, while *N* is the number of observations. The optimal value of RMSE is 0, which denotes a perfect match of the model. RE is expressed as a percentage with a lower value indicating a better fitness.

Unlike Adj.*r*^2^ and RMSE that evaluate the performance of the fitting model, RE measures the error of the fitted soil moisture content at a specified soil water potential. A paired-*t* test was performed to compare the fitness of these models and to analyze their indices.

## Results

### Changes in SWRCs with degradation

**Figure 3:**
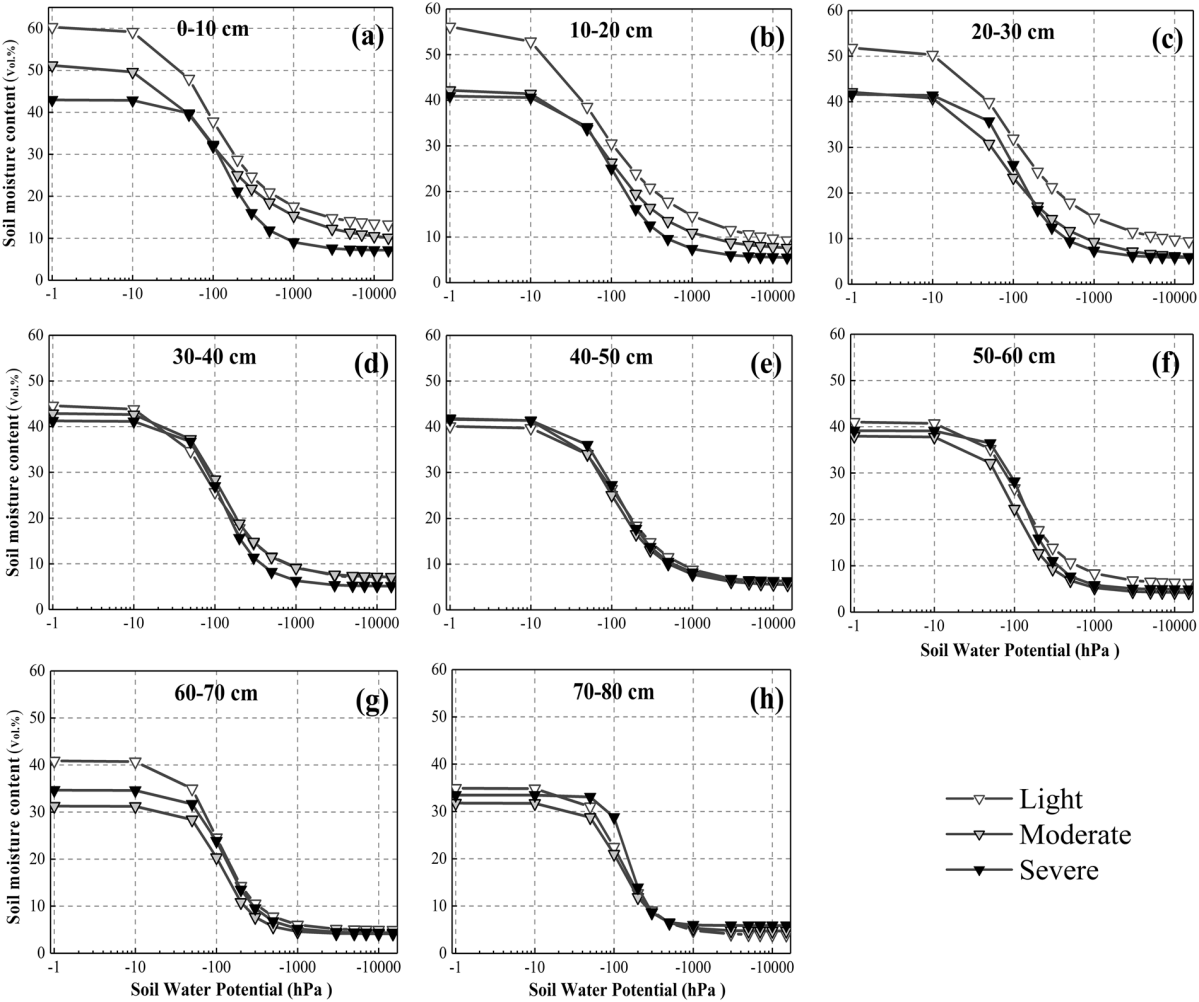
SWRCs of 0 cm–80 cm soil depths for all degradation degrees.

Before further analysis, the 13 scatter points of each soil sample were connected to see the general change patterns of SWRCs (Fig. 3). The alpine meadow degradation showed a significantly negative effect on SWRCs. The soil water content decreased along with alpine meadow degradation at almost all soil water potentials for each soil depth, and the SWRCs seem to “move downward” integrally. By contrast, the soil water potential corresponding to the same soil water content increased along with alpine meadow degradation. From light to severe degradation, the mean soil water content across the whole potential section decreased by 31.1%, 29.9%, 27.5%, 9.6%, 1.7%, 8.2%, 11.2% and 1.8% for the 0–10, 10–20, 20–30, 30–40, 40–50, 50–60, 60–70, and 70–80 cm depth layers, respectively. Such decreasing trend was most obvious in the 0–30 cm layer, thereby indicating that the degradation mainly exerts its influence in the top soils. The SWRCs also became lower with soil depth, and the shape of these curves did not show any significant changes along with degradation and depth, with each curve decreasing slightly then sharply until reaching stability.

**Figure 4:**
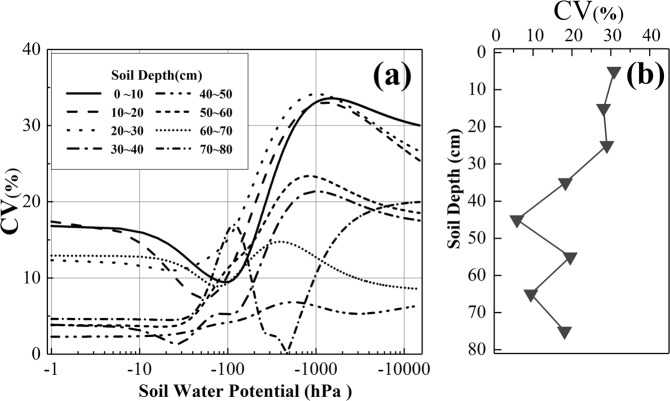
Distribution of CV with changes in soil depth and soil water potential.

The coefficient of variance (CV, variance divided by the mean value) denotes the difference of SWRCs across various degradation degrees. Fig. 4a showed that the CV of soil moisture content in all soil horizons has fluctuated remarkably yet showed an increasing trend along with decreasing soil water potential. The CV of SWRCs across different degradation degrees decreased along with soil depth (Fig. 4b), and the CV of the 0 cm–30 cm soil depth was higher than 20%, thereby indicating that the effect of degradation on SWRCs was mainly manifested in the top soils.

### Comparison of model fitness

**Figure 5:**
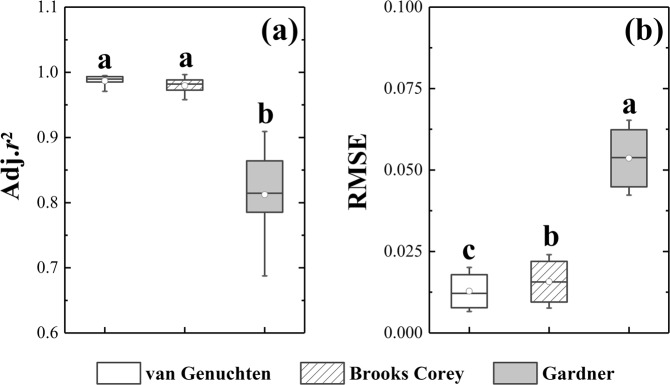
Adj.r^2^ and RMSE of different models. The different letters on the error bars denote a significant difference (p < 0.05) among the three models.

**Figure 6:**
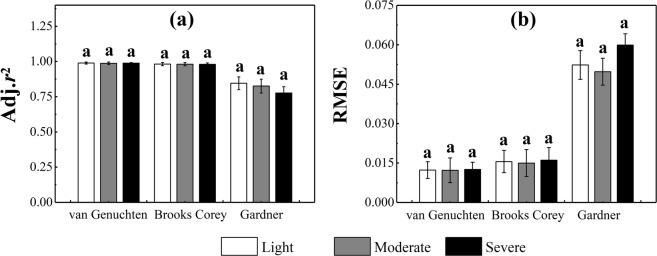
Adj.r^2^ and RMSE of different degradation degrees for each model. The letters above the bars indicate no significant difference among the three models.

Tables 3 and 4 show the Adj.*r*^2^ and RMSE of the van Genuchten, Brooks Corey, and Gardner models. The Adj.*r*^2^ values of these models ranged from 0.971 to 0.995, 0.958 to 0.997, and 0.688 to 0.909, respectively, while their RMSE values ranged from 0.007 to 0.020, 0.008 to 0.024, and 0.042 to 0.063, respectively. The paired-t test showed that the Adj.*r*^2^ values of both the van Genuchten and Brooks Corey models were significantly higher than that of the Gardner model (*p* < 0.05) (Fig. 5a), while the RMSE of the Gardner model was significantly higher than those of the van Genuchten and Brooks Corey models. Unlike Adj.*r*^2^, the RMSE values of the van Genuchten and Brooks Corey models showed a significant difference (Fig. 5b). By combining these two indices together, the van Genuchten and Brooks Corey models were deemed sufficiently accurate and were much better than the Gardner model in terms of fitting the SWRCs of an alpine meadow. Furthermore, the Adj.*r*^2^ and RMSE of each model did not vary along with degradation degree (Fig. 6), thereby indicating that degradation does not affect the fitness of each model.

**Table 3:** Adj.r^2^ of different fitting models.

Soil depth (cm)	van Genuchten			Brooks Corey			Gardner		
	LD	MD	SD	LD	MD	SD	LD	MD	SD
0–10	0.994	0.991	0.985	0.997	0.989	0.974	0.869	0.886	0.783
10–20	0.995	0.976	0.982	0.990	0.970	0.971	0.909	0.863	0.828
20–30	0.991	0.987	0.990	0.985	0.981	0.981	0.883	0.879	0.805
30–40	0.979	0.985	0.985	0.971	0.977	0.966	0.869	0.823	0.791
40–50	0.977	0.971	0.986	0.963	0.958	0.978	0.840	0.829	0.812
50–60	0.994	0.995	0.992	0.988	0.988	0.986	0.817	0.795	0.754
60–70	0.992	0.995	0.995	0.982	0.993	0.992	0.786	0.749	0.748
70–80	0.989	0.993	0.985	0.973	0.985	0.992	0.787	0.779	0.688

**Table 4:** RMSE of different fitting models.

Soil depth (cm)	van Genuchten			Brooks Corey			Gardner		
	LD	MD	SD	LD	MD	SD	LD	MD	SD
0–10	0.011	0.012	0.015	0.008	0.013	0.024	0.058	0.046	0.063
10–20	0.010	0.017	0.015	0.013	0.019	0.020	0.045	0.044	0.053
20–30	0.012	0.012	0.012	0.015	0.015	0.017	0.049	0.042	0.058
30–40	0.018	0.014	0.015	0.021	0.018	0.023	0.048	0.055	0.062
40–50	0.017	0.020	0.013	0.021	0.024	0.017	0.049	0.054	0.056
50–60	0.009	0.008	0.011	0.012	0.012	0.014	0.054	0.056	0.065
60–70	0.011	0.007	0.007	0.016	0.008	0.010	0.061	0.052	0.058
70–80	0.011	0.008	0.013	0.017	0.011	0.009	0.054	0.048	0.063

### Fitting error of the van Genuchten and Brooks Corey models

Section “Comparison of model fitness” has revealed that the Gardner model fits the SWRCs of a degraded alpine meadow poorly compared with the van Genuchten and Brooks Corey models. Therefore, the Gardner model is excluded from the following analysis.

**Figure 7:**
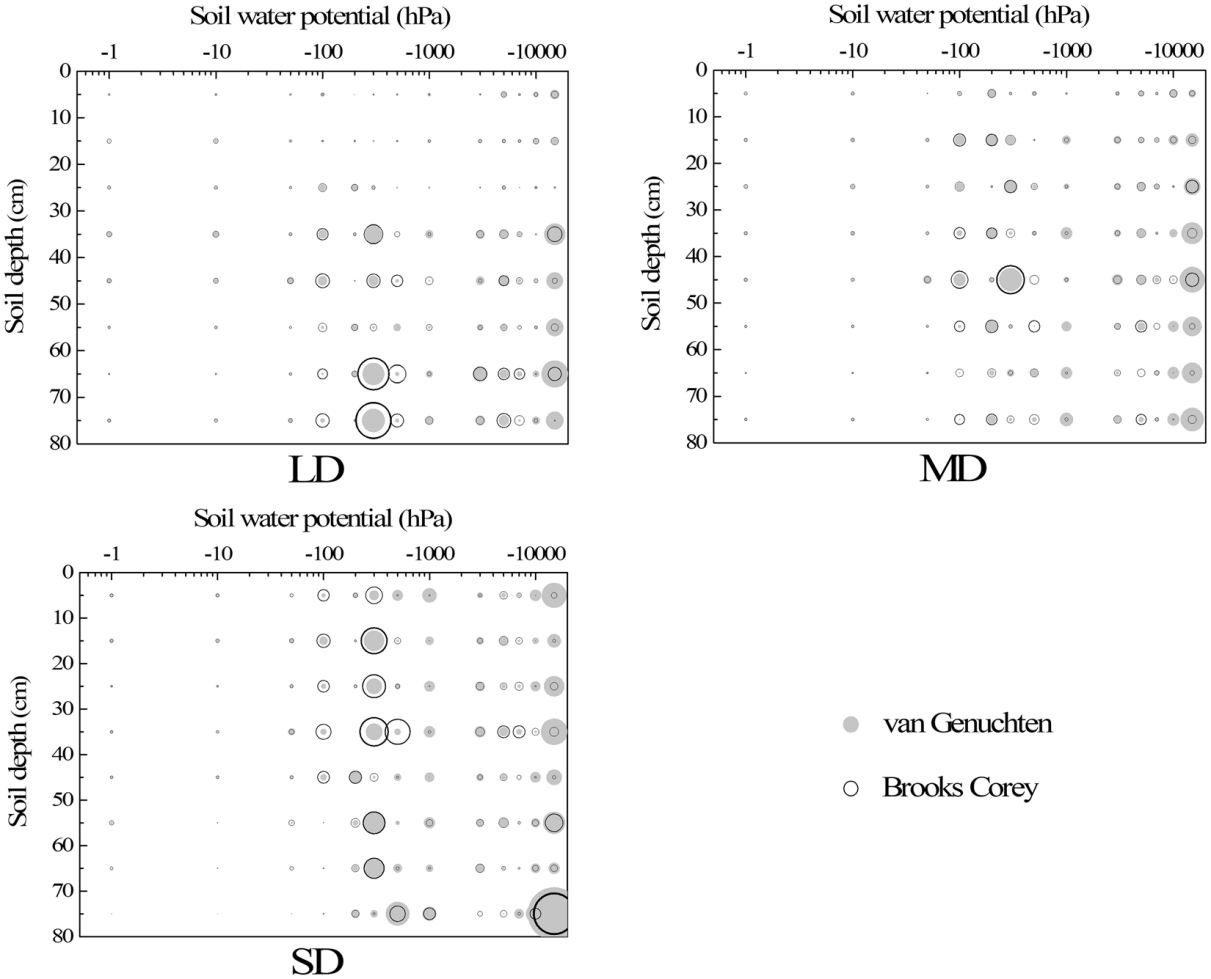
RE distribution of different models for each degradation degree. A smaller circle (dot) indicates a lesser error.

For nearly all degradation degrees and soil depths, the fitting errors of the two models were mainly distributed in the higher (from –100 hPa to –500 hPa) and lower (below –10000 hPa) sections of soil water potential (Fig. 7). The fitting errors in the middle section (from –500 hPa to –5000 hPa) were relatively minor, while those from 0 hPa to –100 hPa were minimal for the measured saturated water content that was put into use. The fitting errors of the whole curve were negligible for those soils at the 0 cm–30 cm layer of LD and 0 cm–10 cm layer of MD. Similar to Adj.*r*^2^ and RMSE, the fitting errors between the van Genuchten and Brooks Corey models showed no significant differences and did not vary along with degradation.

### Key parameters of the van Genuchten and Brooks Corey models

The van Genuchten and Brooks Corey models have six parameters, among which *θ*s, *θ*r, curve shape (*n* and *λ* for the van Genuchten and Brooks Corey models), and air entry pressure (1/α for the van Genuchten and *h*d for the Brooks Corey model) were built in, while field capacity and permanent wilting moisture were derived from the fitting curves. The soil water content at field capacity usually corresponds to a soil water potential between –100 hPa and –340 hPa, while the permanent wilting moisture has a corresponding soil water potential of approximately –15000 hPa.

**Figure 8:**
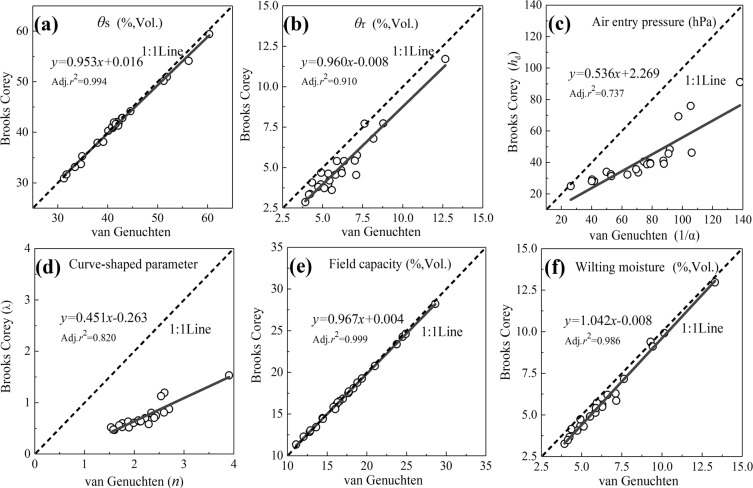
Comparison of key parameters between the van Genuchten and Brooks Corey models.

The comparisons of key parameters between the van Genuchten and Brooks Corey models are showed in Fig. 8. For *θ*s, field capacity, and permanent wilting moisture, the two models showed a satisfactory coherence with Adj.*r*^2^ values of higher than 0.98. The *θ*r, curve shape parameter, and air entry pressure of Brooks Corey model were much lower than those of the van Genuchten model, and both models showed a relatively poor coherence with Adj.*r*^2^ values ranging from 0.74 to 0.91.

## Discussion

### Effect of degradation on the SWRCs of an alpine meadow and the consequent hydrological implications

SWRC is subject to various soil properties such as bulk density, organic carbon, soil texture and aggregate size^44^. In our study, soil properties change drastically with increasing degradation degrees (Table 2), thereby resulting in the variations of SWRCs (Fig. 3). A reduction in soil organic carbon and clay content and an increase in bulk density and sand content directly lead to low porosity, low water holding capacity, and integral downward movement of SWRCs for most soil layers. These phenomena have been confirmed and need no repetition^28,38^.

Table 2 shows that the basic properties of the lower soil layers (below 30 cm) in the experimental field are relatively consistent. Therefore, the differences in the SWRCs across different degradation degrees were mainly manifested in the upper soil layers (0 cm to 30 cm), and CV decreased along with soil depth (Fig. 4b). The low soil water content at a low soil water potential (<−1000 hPa) magnified the variances across different degradation degrees, and CV was relatively higher in the lower section (Fig. 4a). Unlike the higher section (>−1000 hPa) where gravity is dominating, the soil water content at the lower section was mainly trapped by the capillary effect and absorbed by soil particles, that is, the differences in the soil properties across various degradation degrees were fully manifested at the lower section^11,45^.

Despite the overall decrease in soil water content from 0 hPa to 15000 hPa, the curve shape of all measured SWRCs did not change significantly along with degradation and depth. Therefore, no significant differences were observed in the fitness of the models across different degradation degrees. Fig. 3 shows that most soils are sandy, while the SWRCs for non-saline soils largely depend on soil texture^46^. Therefore, curve shape did not vary significantly unlike soil water content. Similar conclusions were also obtained in many other studies^29,38^.

As the direct implication of SWRCs, the soil water holding capacity decreased along with degradation. From the energy perspective, the soil water potential corresponding to soil water content increased along with degradation (Fig. 3), thereby indicating that degradation not only reduced soil water content but also facilitated soil water loss. Nevertheless, the soil water potential increased along with degradation, thereby facilitating root uptake.

### General applicability of the van Genuchten and Brooks Corey models and the limitations of the Gardner model

The van Genuchten and Brooks Corey models have been widely accepted in the literature^13,14^. Our study validated the reliability of these models in fitting the SWRCs of degraded alpine meadow soil on the eastern Tibetan Plateau, thereby broadening their scope of application. The fitness of these models depends on the shape of SWRCs, which are mostly controlled by air entry pressure^47^. Air starts to penetrate into the soil pores when the pressure exceeds the air entry pressure, and then the soil water discharges with acceleration^14^. This parameter has also been closely associated with pore structure, with a lower value indicating the presence of more macropores in soil^48^. A wide range of soils from clay to sand have detectable air entry pressure, but the air entry pressure for some coarse, stony soil with extremely large pores may approach zero^49^.

**Figure 9:**
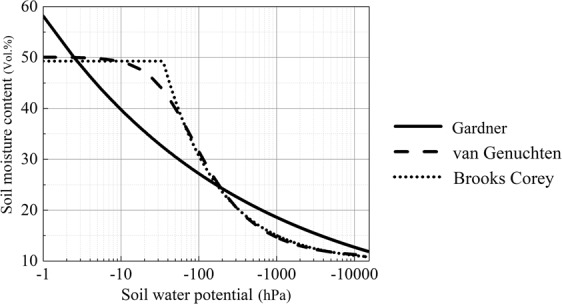
Curve shapes of the three models.

Fig. 9 shows the curve shapes of the three models. Both the van Genuchten and Gardner models are smooth and continuous, while the Brooks Corey model is a piece-wise equation with a break point at air entry pressure. The descending trends of these curves in the lower potential section (<−100 hPa) tend to coincide, and the van Genuchten and Brooks Corey models are nearly congruent except for the neighboring section around the air entry pressure. Therefore, no differences is observed in the fitness of these two models, and the error distribution of both models tends to be consistent.

As a simple power function without a distinct turning point to represent the air entry pressure value, the Gardner model remarkably contrasts the other two models, especially in the higher potential section between 0 hPa and –100 hPa (Fig. 9). Compared with the van Genuchten and Brooks Corey models, the Gardner model has a significantly lower accuracy for fitting the SWRCs of a degraded alpine meadow. This was consistent with the results of early studies^31^. However, this model is still being used in some cases because of its simplicity^50,51^. Wen *et al*.^52^ and Wang *et al*.^29^ used the Gardner model to fit the SWRCs of alpine meadows with different degrees of degradation in the Tibetan Plateau, and both studies reported the favorable fitness of this model with an average *r*^2^ value of 0.99. Our conclusions seem to contradict their findings, but such contradictions are explainable. The experimental field in Wen *et al*.^52^ is located in the source area of the Yangtze River with an elevation of higher than 4600 m and with a pedogenesis retarded by extremely low temperature and lack of vegetation. Their soil samples comprise a large fraction of coarse sand and gravel that belong to rhogosol without a detected air entry pressure. By contrast, the measuring points in the experiment of Wang *et al*.^29^ are initiated with –100 hPa. Figure 9 showed that the Gardner model tends to coincide with the two other models. Therefore, the Gardner model also favorably fits the measuring points of the two studies. In addition, the van Genuchten model was reported to be not capable for fitting the SWRCs of soil with uneven pore size distribution^31^, which is not reflected in our study.

The SWRCs depends not only on the wetting or drying pathway of the soil, but also on the size and shape of particles, and the porosity of the soil^53^. The applicability of models for SWRCs may be limited due to the high spatial heterogeneity of soil properties, thus it is significant and necessary to compare these models to analyze soil moisture dynamic and soil hydrology. Given the importance and irreplaceability of soil water conservation function of alpine meadow in Tibetan Plateau, our findings will provide a more comprehensive understanding about the soil moisture dynamic and the soil hydrological effects of vegetation degradation to guarantee water supply and ecological security.

## Conclusion

This study used the van Genuchten, Brooks Corey and Gardner models to fit the SWRCs of LD, MD and SD alpine meadows on the eastern Tibetan Plateau. The fitness, error distribution and key parameters of these models were also compared. The results showed that the soil moisture content of all soil horizons at different soil water potentials ranging from 0 hPa to –15000 hPa decreased unanimously with degradation, thereby pushing the SWRCs downward. Moreover, the differences in the SWRCs across varying degradation degrees decreased along with soil depth. Both the van Genuchten and Brooks Corey models were applicable for fitting the SWRCs of alpine meadows with different degrees of degradation, while the Gardner model showed a relatively poor performance. Regardless of degradation degree and soil depth, the errors of the van Genuchten and Brooks Corey models were mainly distributed in the higher (from –100 hPa to –500 hPa) and lower (below –10000 hPa) potential sections. The key parameters, including *θ*s, field capacity, and permanent wilting moisture, of the van Genuchten model were highly coherent with those of the Brooks Corey model, while the *θ*r, curve shape parameter, and air entry pressure of the Brooks Corey model were lower than those of the van Genuchten model. Alpine meadow degradation has a significant negative effect on soil water holding capacity, especially for the upper soil layers (0 cm to 30 cm), but the shape of the SWRCs does not significantly change along with degradation.

## Data Availability

All data generated or analysed during this study are included in this published article.
